# Inactivation of hypoxia-induced YAP by statins overcomes hypoxic resistance tosorafenib in hepatocellular carcinoma cells

**DOI:** 10.1038/srep30483

**Published:** 2016-08-01

**Authors:** Tian-yi Zhou, Lin-han Zhuang, Yan Hu, Yu-lu Zhou, Wen-kai Lin, Dan-dan Wang, Zi-qian Wan, Lin-lin Chang, Ying Chen, Mei-dan Ying, Zi-bo Chen, Song Ye, Jian-shu Lou, Qiao-jun He, Hong Zhu, Bo Yang

**Affiliations:** 1 Zhejiang Province Key Laboratory of anti-cancer Drug Research, Institute of Pharmacology and Toxicology, College of Pharmaceutical sciences, Zhejiang University, Hangzhou, China; 2 First Affiliated Hospital, School of Medicine, Zhejiang University, Hangzhou, China

## Abstract

Sorafenib is a multikinase inhibitor used as a first-line treatment for advanced hepatocellular carcinoma (HCC), but it has shown modest to low response rates. The characteristic tumour hypoxia of advanced HCC maybe a major factor underlying hypoxia-mediated treatment failure. Thus, it is urgent to elucidate the mechanisms of hypoxia-mediated sorafenib resistance in HCC. In this study, we found that hypoxia induced the nuclear translocation of Yes associate-Protein (YAP) and the subsequent transactivation of target genes that promote cell survival and escape apoptosis, thereby leading to sorafenib resistance. Statins, the inhibitors of hydroxymethylglutaryl-CoA reductase, could ameliorate hypoxia-induced nuclear translocation of YAP and suppress mRNA levels of YAP target genes both *in vivo* and *in vitro*. Combined treatment of statins with sorafenib greatly rescued the loss of anti-proliferative effects of sorafenib under hypoxia and improved the inhibitory effects on HepG2 xenograft tumour growth, accompanied by enhanced apoptosis as evidenced by the increased sub-G1 population and PARP cleavage. The expression levels of YAP and its target genes were highly correlated with poor prognosis and predicted a high risk of HCC patients. These findings collectively suggest that statins utilization maybe a promising new strategy to counteract hypoxia-mediated resistance to sorafenib in HCC patients.

Hepatocellular carcinoma (HCC) is one of the most common malignant solid tumours worldwide. Most HCC patients are at an advanced stage at the time of diagnosis and can only be treated with chemotherapy due to the low response to hepatic resection and liver transplantation therapy^1^. Sorafenib, a multikinase inhibitor, is the first and only drug that is clinically approved for patients with advanced HCC^2^. Sorafenib has dual mode of action:1) interfering with multiple receptor tyrosinekinases(VEGFR, PDGFR) to inhibit tumour angiogenesis; 2) blocking the Raf/MEK/ERK signalling pathway to suppress cancer cell proliferation^3^,^4^. However, the use of sorafenib to treat late-stage HCC patients has been challenged by the fact that the overall survival benefit is limited to months (2–3 months), which maybe attributable to the intrinsic differences in the response of HCC cells to sorafenib: some HCC tumours are primarily resistant to sorafenib, while others develop resistance after an initial response^2^,^5^,^6^. Several lines of evidences have shown that the intratumour hypoxia of HCC is highly correlated with the compromised efficacy of sorafenib in HCC patients, but the underlying mechanisms remain elusive^7^,^8^.

As one of the most conspicuous characteristics of HCC, intratumour hypoxia occurs in the HCC microenvironment when the oxygen supply from the bloodstream is not sufficient to support the outgrowth of HCC cells^9^. As a result, hypoxia-triggered angiogenesis becomes abnormally active; in this context, therapeutic strategies to counteract angiogenesis are applied as a mono-treatment or in combination with chemotherapeutic agents to eliminate the hypoxia-induced malignancy of HCC. To this end, the antiangiogenic effect of sorafenib should benefit those HCC patients suffering from tumour hypoxia^10^. However, recent unexpected reports have indicated that sorafenib treatment may aggravate the intratumour hypoxia by excessive vessel pruning, which reciprocally promotes subsequent therapeutic resistance^10^,^11^.

Hypoxia-inducible factor 1α (HIF-1α), a pivotal regulator of the cellular response to hypoxia, is believed to be responsible for sorafenib resistance in hypoxic environments^7^,^10^. Nevertheless, HIF-1α-targeting strategies may not be the best way to reverse sorafenib resistance because no HIF-1α inhibitor has been clinically approved to treat cancer. Thus, the exploration of alternative key factors underlying the hypoxia-mediated sorafenib resistance could provide an efficient way to overcome resistance. In addition, the key factor(s) that plays a critical role in hypoxic resistance to sorafenib should be assessed for the clinical availability of potential inhibition strategies.

The Yes-associated protein (YAP), a transcriptional co-activator and a potent growth promoter in the Hippo pathway, has been found to play an important role in tumourigenesis, development, and metastasis^12^,^13^. It has been shown that YAP expression is highly associated with advanced stage and poor prognosis in HCC^14^,^15^, which is also validated in our present study. Intriguingly, a recent study revealed the nuclear translocation of YAP under hypoxia^16^. In our previous study, we found that YAP nuclear translocation primed by hypoxia may resulted in the resistance towards a Topoisomerase I inhibitor irinotecan^17^. However, irinotecan is not among the most-widely clinical used drugs to treat HCC, thus it would be more interesting to investigate whether hypoxia-activated YAP is involved in the resistance of sorafenib, the first-line drug for the treatment of advanced HCC. Particularly, given the fact the sorafenib possessing the anti-angiogenetic effects *in vivo*, it generally resulted in aggravated intratumour hypoxia. In the present study, we showed that YAP nuclear localization was significantly increased in both hypoxic HCC cells and xenograft tumours, along with increased mRNA levels of the YAP target genes *AREG, CTGF* and *Cyr61*. Silencing of YAP with small interfering RNA (siRNA) sensitized the cells to sorafenib under hypoxia, thus highlighting the contribution of YAP to hypoxia-mediated sorafenib resistance.

Statins are a class of specific inhibitors of 3-hydroxymethylglutaryl-CoA reductase (HMGCR), an enzyme that catalyses the production of mevalonate in the mevalonate pathway for the biosynthesis of isoprenoids^18^. Statins have been clinically used to lower cellular cholesterol levels in patients with hypercholesterolemia^19^. Recently, the anti-cancer effects of statins have been reported, although the mechanisms of action are still undetermined^20^. Sorrentino *et al*. showed that statins reduced YAP nuclear localization and inhibited YAP transcriptional activity, regulating cell proliferation and self-renewal^21^. These studies examined the effects of statins under normoxia, and given the link between YAP nuclear localization and hypoxic HCC, we were prompted to investigate whether statins were able to interfere with the activation of YAP induced by hypoxia. Interestingly, we found that statins significantly rescued hypoxia-mediated loss of YAP phosphorylation on S127 as well as the transactivation of YAP target genes. Further studies showed that statins could overcome the hypoxic resistance of HCC cells to sorafenib *in vitro* and sensitized HepG2 xenografts to sorafenib *in vivo* by preventing the nuclear accumulation and activation of YAP.

## Results

### YAP activation contributes to hypoxia-mediated sorafenib resistance in HCC cells

**Figure 1:**
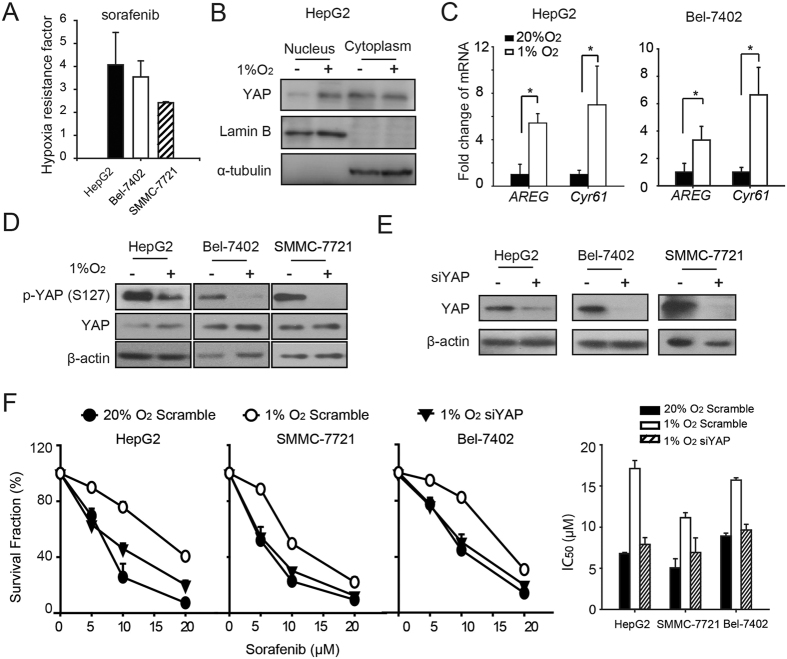
YAP activation is correlated with hypoxia-mediated sorafenib resistance. (**A**) The hypoxic resistance factor of sorafenib in three HCC cell lines, HepG2, Bel-7402, and SMMC-7721,was calculated as IC_50_-hypoxia/IC_50_-normoxia. Data are representative of 3 independent experiments and are expressed as the mean ± SD. (**B**) The protein level of YAP was determined in the nucleus and cytoplasmic fractions by western blot assays. HepG2 cells were cultured under normoxic or hypoxic conditions for 24 h. (**C**) Quantitative RT-PCR (qRT-PCR) analysis of YAP target genes in HepG2 and Bel-7402 cells. Cells were exposed to normoxia or hypoxia for 24 h. Data are representative of 3 independent experiments and are expressed as the mean ± SD. *P < 0.05, ***P < 0.001. (**D**) The protein level of YAP and YAP-S127 phosphorylation were detected by western blot analysis in three HCC cell lines, HepG2, Bel-7402, and SMMC-7721. Cells were cultured under normoxia or hypoxia for 24 h. (**E**) The protein level of YAP was detected by western blotting after silencing YAP in HCC cell lines under hypoxia. (**F**) Cell viability and the IC_50_ of sorafenib were determined in YAP-silenced or control cells after sorafenib treatment (48 h) by SRB assays.

The high correlation between hypoxia and sorafenib resistance has been described in previous studies^10^,^22^. Similar observations were also noted in the present study: the hypoxic resistance factor of sorafenib (calculated as: IC_50_ values under hypoxia/IC_50_ under normoxia)in the 3 HCC cell lines HepG2, Bel-7402 and SMMC-7721 were 4.07, 3.54 and 2.41, respectively (Fig. 1A). Given that recent evidence indicated that hypoxia inhibits LATSs, thus promoting YAP activation^16^, we were prompted to investigate the contribution of YAP to hypoxia-mediated resistance to sorafenib. Firstly, we examined whether YAP was activated by hypoxia in HCC cells. Our results showed that in HCC cells, hypoxia exposure increased nuclear accumulation of YAP (Fig. 1B), accompanied by the reduced YAP-S127 phosphorylation (Fig. 1D). Consequently, the mRNA levels of YAP target genes, including *AREG* and *Cyr61*, were increased upon hypoxia, for instance, in Bel-7402 cells, the fold change of these genes compared to expression in normoxic conditions was 4.44 and 5.37, respectively. To establish a link between the YAP activation and sorafenib resistance under hypoxia, YAP was depleted by siRNA under hypoxia prior to the sorafenib treatment. YAP deletion under hypoxia (Fig. 1E) significantly reduced the surviving fraction of sorafenib-treated cells, as indicated by the decreased IC_50_ values under hypoxia (Fig. 1F).

### Atorvastatin inhibits YAP nuclear translocation in HCC cell lines and HepG2 xenograft tumours

**Figure 2:**
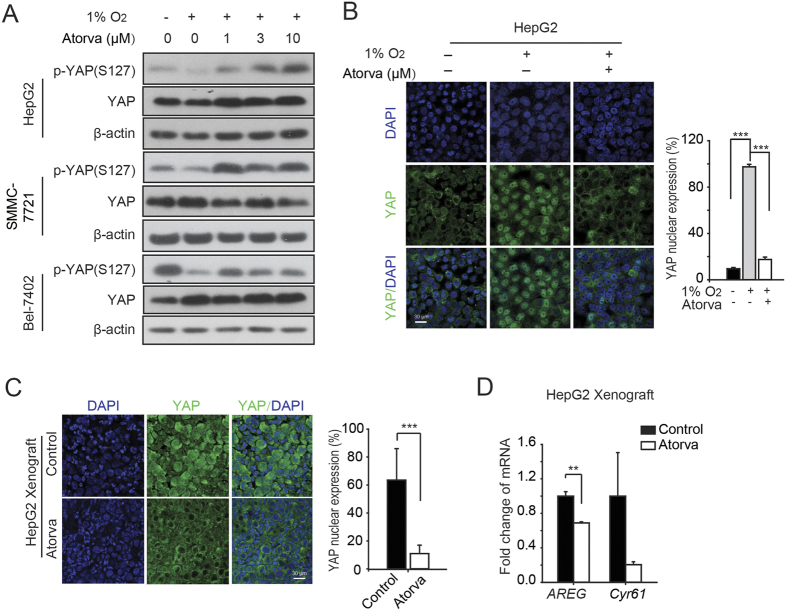
Statins inhibit YAP transcriptional activity under hypoxia both *in vitro* and *in vivo.* (**A**) The protein level of phosphorylated YAP-S127 was detected by western blot analysis. Three HCC cell lines, HepG2, Bel-7402, and SMMC -7721, were treated with atorvastatin (1, 3, 10 μM) under hypoxia for 24 h.(**B**) Representative images of immunofluorescence analysis of YAP in HepG2 cells. Cells were treated with or without atorvastatin (10 μM) under hypoxia for 24 h. Scale bar, 30 μm. The percentage of nuclear YAP population of each groups was displayed. (**C**) Representative images of immunofluorescence analyses of YAP in atorvastatin-treated HepG2 xenograft tumour tissues. The nuclear YAP population of control and atorvastatin groups was displayed.(**D**) The expression of the YAP target genes *AREG* and *Cyr61* was determined by quantitative PCR (qRT-PCR) analysis in HepG2 xenograft tumour tissues. Data are representative of 3 independent experiments and are expressed as the mean ± SD.

The previous data revealed that hypoxia-activated YAP may play an important role in hypoxia-induced sorafenib resistance; thus, the prevention or inhibition of YAP activation under hypoxia could be an efficient way to interfere with the hypoxic resistance to sorafenib, improving the anti-cancer efficacy of sorafenib against HCC. In addition to the enhanced ability to kill HCC cells, the tolerance and safety of drug candidates should not be compromised when pursuing strategies to combat hypoxia-mediated resistance to sorafenib in HCC cells. Several lines of evidence have indicated that cervastatin and simvastatin are capable of suppressing the nuclear translocation of YAP^21^,^23^. However, the suppressive effects by statins were achieved under normoxic conditions. Considering the diverse mechanisms underlying the nuclear accumulation of YAP following different stimuli, it would be interesting to determine whether statins could also interrupt the hypoxia-induced YAP activation. Figure 2A shows that atorvastatin effectively rescued the hypoxia-triggered loss of YAP-S127 phosphorylation, which generally indicates the cytoplasmic retention of YAP. These data indicate that the nuclear YAP activity was reduced. Furthermore, immunofluorescence staining was utilized to determine the nuclear or cytoplasmic localization of YAP. Under hypoxia, nuclear YAP was observed in HepG2 cells, whereas the exposure of hypoxic cells to atorvastatin strongly attenuated the nuclear accumulation of YAP (Fig. 2B), supporting the idea that statins alter YAP activity induced by the hypoxic environment *in vitro*. In addition to the cellular model, the IF analyses of HepG2 xenograft tumours treated with atorvastatin showed that YAP was predominantly distributed in the cytoplasm compared with that in the vehicle groups (Fig. 2C). We also monitored the effect of atorvastatin on YAP target gene (*AREG, CYR61*) expression in tumours by qRT-PCR. As shown in Fig. 2D, atorvastatin inhibited YAP transcriptional activity and target gene expression in HepG2 xenograft tumours. These findings demonstrate that statins inhibit YAP nuclear accumulation under hypoxic conditions, thus potentially enhancing the efficacy of sorafenib in HCC.

### A combination treatment of sorafenib and statins has more potent anti-proliferative effects on HCC cells under hypoxia

**Figure 3:**
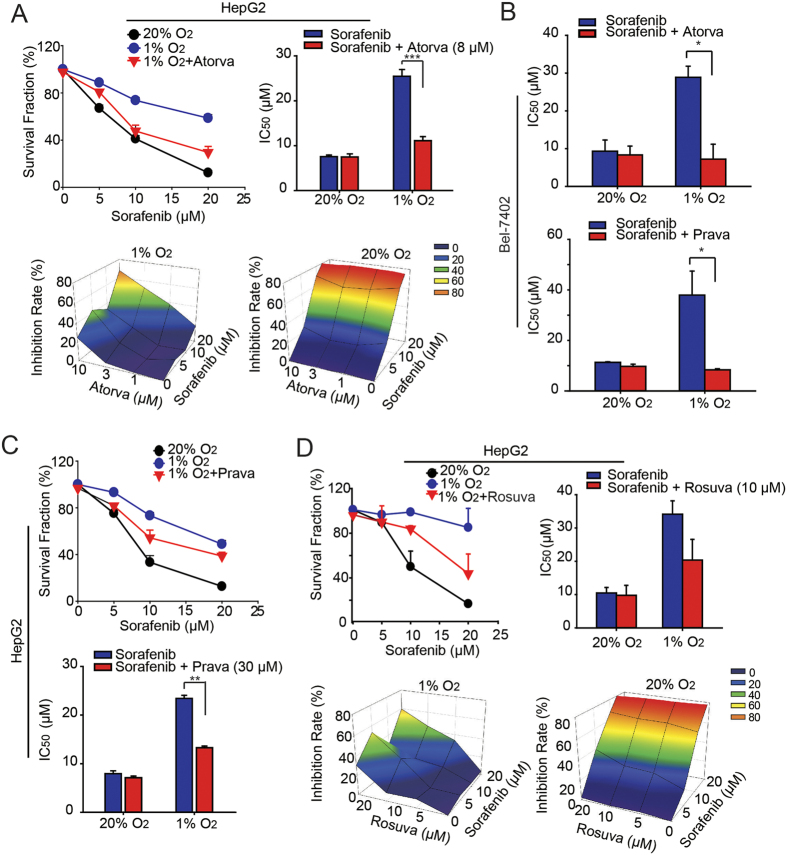
Hypoxia-induced sorafenib resistance can be overcome by statins *in vitro*. (**A**,**C**,**D**) HepG2 cells were incubated with sorafenib at serial concentrations (48 h) in the presence or absence of atorvastatin (8 μM), pravastatin (30 μM) or rosuvastatin (10 μM) at different concentrations, and the cell viability and IC_50_ values were then determined by SRB assays. Data are representative of 3 independent experiments and are expressed as the mean ± SD. (**B**) Bel-7402 cells were treated with sorafenib at various serial concentrations (48 h) in the presence or absence of atorvastatin or pravastatin, and the IC_50_ values of sorafenib were determined by SRB assays. Data are representative of 3 independent experiments and are expressed as the mean ± SD. “Heat” graphs were presented to display the combination effects. Z-axis: Inhibition ratio of the treated groups under hypoxia and normoxia; X-axis: the concentration of statins; Y-axis: the concentration of sorafenib. And the colors representative for the inhibition ratio were presented. Blue: 0–20% inhibition, green: 40% inhibition, yellow: 60% inhibition, red: 80% inhibition.

As previously described in the current study, 1) nuclear translocation of YAP may confer hypoxia-induced resistance on HCC cells; 2) statins are capable of inhibiting YAP nuclear accumulation and transcriptional activity induced by hypoxia. Therefore, we were interested in determining whether statins can overcome hypoxia-mediated resistance to sorafenib by interfering with YAP activation. As shown in Fig. 3A, atorvastatin treatment (48 h) greatly enhanced the anti-cancer effect of sorafenib compared with that under hypoxia, as indicated by the decreased IC_50_ values from 25.5 μM to 10.9 μM after the combination treatment in hypoxic conditions. Intriguingly, in contrast to hypoxia, in normoxic conditions, atorvastatin had minimal effects on the cytotoxicity caused by sorafenib, further indicating that YAP was particularly critical for HCC cells under hypoxia. Similar combinatorial effects were also observed in Bel-7402 cells (Fig. 3B) or with pravastatin (Aladdin,81131-70-6) or rosuvastatin (Aladdin, 147098-20-2) (Fig. 3C,D).

### Statins enhance sorafenib-induced apoptosis under hypoxia by suppressing YAP target genes

**Figure 4:**
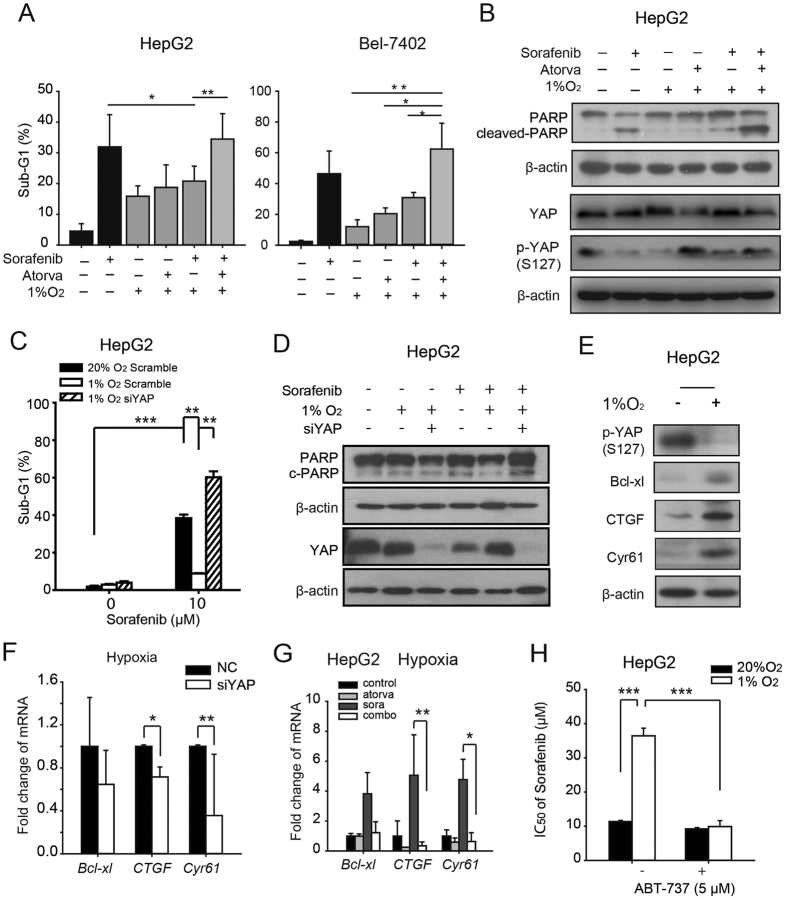
Statins increase sorafenib-induced apoptosis under hypoxia by suppressing YAP target genes. (**A**) The percentage of apoptotic cells was measured by PI staining. Bel-7402 cells were treated with sorafenib (10 μM) or atorvastatin (10 μM) alone or both sorafenib (10 μM) and atorvastatin (10 μM) for 48 h under hypoxia. Data are representative of 3 independent experiments and are expressed as the mean ± SD. (**B**) PARP, cleaved-PARP in Bel-7402, HepG2 and SMMC-7721 cells was detected by western blot analysis in the same conditions as (**A**). Data are representative of 3 independent experiments. (**C**) Apoptotic cells following sorafenib treatment (48 h) in YAP-depleted HepG2 cells were measured by PI staining. (**D**) PARP cleavage and YAP protein levels in sorafenib-treated and/or YAP-deleted HepG2 cells were measured. (**E**) qRT-PCR analysis of the YAP target genes *Bcl-xL, CTGF* and *Cyr61* in HepG2 cells. Cells were exposed to normoxia or hypoxia for 24 h. Data are representative of 3 independent experiments and are expressed as the mean ± SD. (**F**) The protein level of phosphorylated YAP-S127 and the YAP target genes *Bcl-xL, CTGF* and *Cyr61* were detected by western blotting in HepG2 cells. Cells were cultured under normoxia or hypoxia for 24 h. (**G**) qRT-PCR analysis of YAP target genes in HepG2 cells after silencing YAP under hypoxia. (**H**) The IC_50_ values of sorafenib combined with vehicle or ABT-737 (5 μM) in HepG2 cells under normoxia or hypoxia. HepG2 cells were incubated with ABT-737 (5 μM) under normoxia or hypoxia for 8 h, then treated with sorafenib at serial concentrations (48 h). ***P < 0.001.

Consistent with the cell viability results, sorafenib-induced apoptosis was significantly reduced by hypoxia, and the loss of apoptotic cells was abolished by atorvastatin in both HepG2 and Bel-7402 cells (Fig. 4A). Sorafenib-induced cleaved-PARP levels were reduced under hypoxia, while a combination of atorvastatin (10 μM) and sorafenib (10 μM) enhanced the protein level of cleaved-PARP while promoted the loss of full length PARP in all the tested 3 HCC cell lines, along with the attenuation of p-YAP loss under hypoxia by atorvastain (Fig. 4B and Figure S3A). In addition, the sensitization effect of atorvastatin on sorafenib-induced apoptosis under hypoxia was mimicked by YAP depletion by siRNA, as shown by the increased sub-G1 population (Fig. 4C) and PARP cleavage (Fig. 4D). However, the depletion of TAZ, a another cofactor that binds to TEADs^24^,^25^, failed to abrogate the hypoxic resistance towards sorafenib, with similar inhibition rates of sorafenib in NC siRNA and TAZ siRNA cells under hypoxia (Figure S1).To further validate the involvement of the YAP target genes in the apoptosis caused by the combination treatment, the target genes with anti-apoptotic functions-*Bcl-xl, CTGF,* and *Cyr61*^26^,^27^,^28^,^29^-were investigated. As shown in Fig. 4E, the mRNA and protein levels of Bcl-xl, CTGF, and Cyr61 were strongly increased under hypoxia, along with decreased phosphorylation of YAP. However, the combination with atorvastatin greatly reduced the mRNA level of *Bcl-xl, CTGF* and *Cyr61* (Fig. 4G). Similar observations were also achieved in those HepG2 cells with depleted YAP by siRNA (Fig. 4H). Then we introduced ABT-737, a small molecule inhibitor against Bcl-xL, to antagonize its anti-apoptotic function, since Bcl-xL is one of the most critical anti-apoptotic YAP target genes. As shown in the Fig. 4H, ABT-737 (5 μM) showed little effects on the activities of sorafenib under normoxia, whereas under hypoxia, ABT-737 significantly rescued the loss of anti-cancer activity of sorafenib caused by hypoxia, as indicated by the decreased IC_50_ values of sorafenib from 36.41 to 9.91 μM. These data indicate that YAP nuclear translocation under hypoxia increased the expression of the anti-apoptotic genes *Bcl-xl, CTGF,* and *Cyr61*, thus inducing sorafenib resistance.

### A combination of atorvastatin and sorafenib improves the tumour growth arrest in HepG2 xenograft models

**Figure 5:**
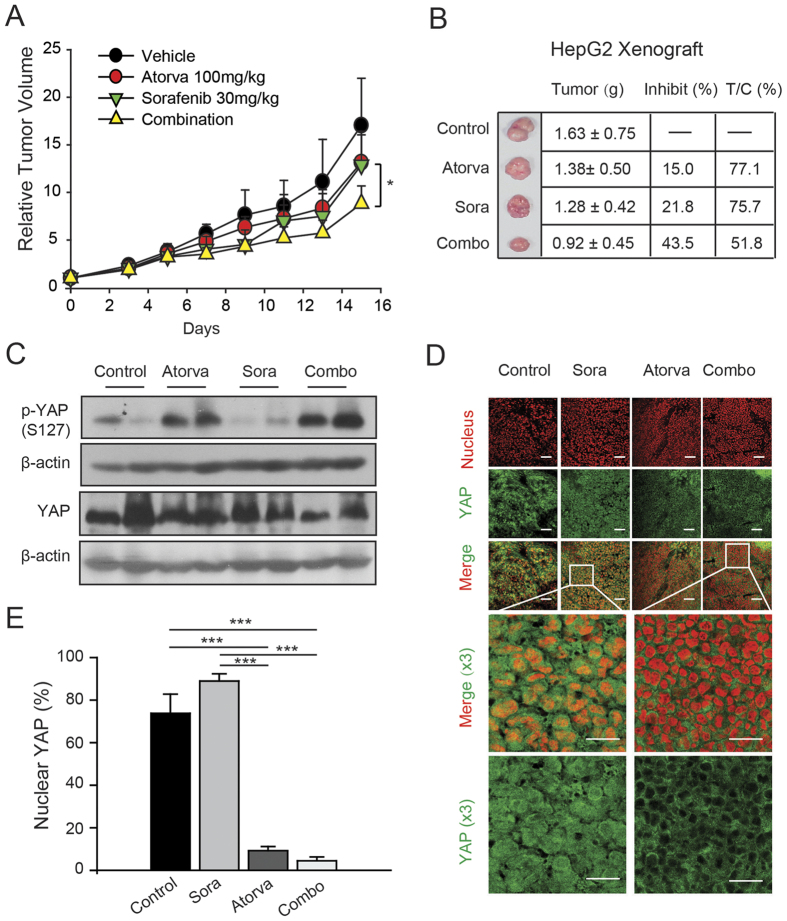
The combination of atorvastatin and sorafenib enhances anti-tumour activities *in vivo*. (**A**) Relative tumour volume of HepG2 xenograft tumour in control, atorvastatin, sorafenib and combination groups. (**B**) Tumour growth inhibition in control, atorvastatin, sorafenib and combination groups. Representative tumours in each group, tumour weight, inhibition ratio and T/C values. (**C**) Phosphorylation level of YAP-S127 in sorafenib- and/or atorvastatin-treated xenograft tumour tissues. (**D**) Immunofluorescence analysis of YAP in xenograft tumours from sorafenib, atorvastatin or combination groups. Scale bars, (up) 50 μm, (bottom) 20 μm. (**E**) Quantification of the nuclear YAP population from the immunofluorescence staining of YAP of the xenograft tumours in each group.

Given the potent anti-cancer effects achieved by combining sorafenib and atorvastatin in HCC cellular models, we next investigated the effects of the combination treatment against HepG2 xenograft models. The combination of sorafenib and atorvastatin had increased inhibitory effects on the xenograft tumour growth, with reduced T/C (77.1%, 75.7% and 51.8% for atorvastatin, sorafenib and combination groups, respectively, Fig. 5A,B) and increased an inhibition ratio (15.0%, 21.8% and 43.5% for atorvastatin, sorafenib and combination groups, respectively, Fig. 5B). We next investigated whether the combination prevented the nuclear accumulation and activation of YAP to establish the causal link between YAP inhibition by atorvastatin and its sensitization of cells to sorafenib. As shown in Fig. 5D,E, YAP was predominantly located in the nucleus of xenograft tumour tissues from vehicle-as well as sorafenib-treated groups. In contrast, atorvastatin in combination with sorafenib effectively excluded YAP from the nucleus. Intriguingly, sorafenib mono-treatment imposed a slightly induction on the nuclear localization of YAP compared with vehicle group, which may owing to the anti-angiogenesis effects and subsequently aggravated caused by sorafenib.Consistent with these findings, sustained sorafenib mono-treatment resulted in a very low level of p-YAP; however, the mono- and combined treatment of atorvastatin led to a remarkable up-regulation of p-YAP (Fig. 5C), indicating the loss of nuclear YAP in these groups. These data collectively indicate that atorvastatin enhanced the anti-cancer activities of sorafenib against HCC xenograft tumours, probably through the inhibitory effects on YAP nuclear accumulation and subsequent activation.

### YAP is associated with poor prognosis of HCC patients

**Figure 6:**
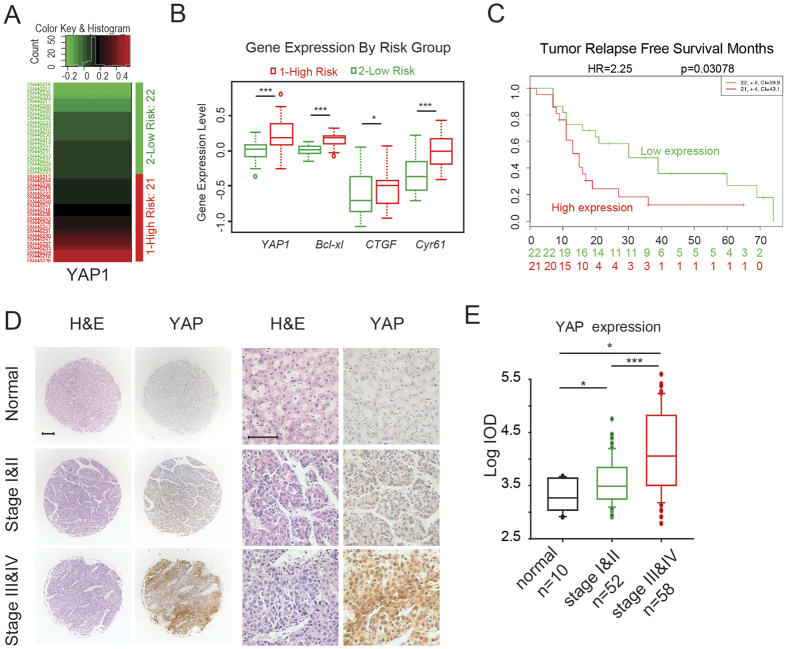
YAP expression is associated with poor prognosis of liver cancer patients. (**A**) Heat map of *YAP* mRNA expression of the liver cancer patient tumour samples along with the risk factor (high and low risk groups were split of the same size depending on the Prognostic Index (risk score) estimated by beta coefficients multiplied by gene expression values. (**B**) Tumour relapse-free survival curve of liver cancer patients with low (n = 22) and high (n = 21) levels of *YAP, Bcl-xl, CTGF* and *Cyr61* expression (*P* < 0.05). (**C**) Gene expression levels of *YAP, Bcl-xl, CTGF* and *Cyr61* in low (n = 22) and high (n = 21) risk groups of liver cancer patients. (**D**) Immunohistochemical staining of YAP in representative normal liver tissues and liver cancer tissues in the tissue microarrays. Scale bars, (left) 50 μm, (right) 200 μm. (**E**) The expression scores of YAP in the tissue microarrays were analysed by Image-Pro Plus. (normal liver tissue: n = 10; stage I &II tumour tissue: n = 52; stage III &IV tumour tissue: n = 58).

To further validate the expression and activation of YAP as a critical clinically relevant factor in HCC patients, we searched the Oncomine database for the expression of YAP in HCC. Three data sets showed a correlation between upregulated YAP expression and HCC patient tumour samples compared with normal human liver samples, and three data sets showed higher levels of YAP in HCC cell lines compared to normal cell lines (Table 1). More importantly, in a data set with 43 HCC cases from the SurvExpress database^30^,^31^, we found that the YAP expression levels were significantly correlated with a higher risk of poor prognosis of the HCC patients (*P* < 0.0001) (Fig. 6A,B), in which those HCC patients with poor prognosis generally harbour higher expression of YAP and its target genes. Intriguingly, the tumour relapse-free survival was significantly longer in HCC patients with lower expression levels of *YAP, Bcl-xl, CTGF,* and *Cyr61 (P* < 0.05) (Fig. 6C).

In addition to the data obtained from these databases, we also conducted immunohistochemical staining of a tissue microarray containing 10 normal liver tissues and 110 liver cancers to further establish the correlation of YAP levels with progression in HCC patients. As shown in Fig. 6D, low-intensity YAP staining was observed in normal liver tissues, while the expression of YAP increased along with the malignancy progression in the HCC tumour samples. Similar results are shown in Fig. 6E, which were determined using log IOD in Image-Pro Plus 6.0, indicating that expression was correlated with tumour stage, as the YAP expression levels from stage III and IV HCC patients were significantly higher than those from stage I and II patients. In addition, we conducted an analysis of the percentage of nuclear YAP in the HCC patient samples on the tissue microarray. As shown in Figure S3, significantly enhanced nuclear YAP was found in stage I and II liver cancer patients, compared to that of normal liver tissues. In addition, a further increment of nuclear YAP percentage was observed in stage III and IV patient samples. Collectively, these data demonstrate that YAP and its target genes (*Bcl-xl, CTGF, Cyr61*) play important roles in the progression of human HCC, and their expression levels were highly correlated with poor prognosis.

## Discussion

Sorafenib is the only standard systematic chemotherapy drug for treatment of advanced HCC. Although clinical studies have shown some survival benefits on the time to progression (TTP) and overall survival (OS), the efficacy of the drug against HCC remains modest, and it usually acts as temporary tumour stabilization^32^. The limited survival benefits with low response rates indicate the existence of drug resistance towards sorafenib among HCC patients.

Recent discoveries of the molecular mechanisms underlying sorafenib resistance have suggested strategies to overcome this issue. Activation of kinase cascades, epidermal growth factor receptor (EGFR) and the PI3K/Akt signalling pathways were found to promote the resistance of HCC cells to sorafenib^33^,^34^. The transition from epithelial to mesenchymal cells promotes the progression of HCC and counteracts the effect of sorafenib^11^,^35^,^36^. All these studies focused on HCC cells and provided evidence that the deregulation of these intrinsic pathways confers resistance on HCC cells towards sorafenib. Based on these efforts, combination therapy of sorafenib with the other anti-cancer agents has been evaluated. A completed multicenter, multinational, randomized phase III clinical study of a combination treatment of sorafenib with erlotinib (SEARCH trial, NCT00901901), a EGFR inhibitor, did not show that the addition of erlotinib to sorafenib met the primary endpoint, and the median OS and TTP were not significantly different in the experimental and control groups^37^, indicating that the inhibition of EGFR may not help sorafenib to improve the survival in patients with advanced HCC.

Because of the intratumour hypoxic microenvironment, which is frequently found in advanced HCC patients, the correlation between sorafenib resistance and hypoxia adaption should be examined. Notably, cancer cells in hypoxic microenvironments often exhibited pro-survival and anti-apoptotic phenotypes, accompanied with resistance to anti-cancer drugs. Importantly, it has been found that sustained sorafenib treatment resulted in decreased microvessel density and aggravated hypoxia, owing to, at least partially, the anti-angiogenesis effects, thus eventually accelerating tumour growth^10^. The levels of intratumour hypoxia might be predictive of HCC response to sorafenib^10^. Considering the failure of most of current chemotherapeutic agents and the strategies targeting solid tumours presenting hypoxia, we were prompted to seek potential novel strategies capable of inhibiting the hypoxia-activated compensatory survival pathways that result in sorafenib resistance.

Accumulating data has revealed a strong correlation between YAP overexpression and carcinogenesis of liver cancer^38^,^39^. Several other studies have shown that YAP may be a predictor of poor prognosis for liver cancer patients^40^, which was confirmed in the present study (Fig. 6). The activated YAP pathway decreased the susceptibility of cancer cells towards a variety of anti-cancer drugs, including RAF and MEK inhibitors^28^, anti-tubulin drugs^41^,^42^, and DNA-damaging agents^42^,^43^. However, whether YAP contributes to the resistance towards sorafenib remains to be investigated. Consistent with previous findings by Ma *et al*.^16^,^17^, our present study showed that in hypoxic HCC cells, YAP was predominantly localized in the nucleus, thus exerting its function, *i.e*., transactivating the target genes *CTGF, Cry61* and others, which may promote hypoxic resistance towards sorafenib (Fig. 1).

Given that the oncogenic functions of YAP in HCC are largely dependent on the nuclear localization^44^, strategies that interfere with the dephosphorylation and nuclear translocation of YAP would be beneficial to overcome sorafenib resistance mediated by hypoxia. Statins, a family of widely used anti-hypercholesterolemic drugs, were shown to have potent suppressive effects on the YAP pathway. Mechanistically, statins increased the phosphorylation of YAP and prevented its nuclear accumulation through the inhibition of HMG-CoA reductase^17^,^21^,^23^. Together with Ma’s findings that hypoxia increased E3 ligase SIAH2 to promote the degradation of LATS2, thus leading to the subsequent reduced p-YAP and activation of YAP^16^, these evidences implicated that hypoxia-activated YAP pathway was a secondary effect, which was regulated by increased levels of HMG-CoA reductase, E3 ligase SIAH2, or some other unidentified factors.

Although mounting evidence has shown the anti-cancer capabilities of statins, their activities under hypoxia remain elusive. In the current study, we demonstrated that statins were capable of: 1) inhibiting the nuclear accumulation of YAP under hypoxia by interrupting its dephosphorylation (Fig. 2), 2)subsequently leading to the restoration of the hypoxia-compromised anti-cancer activity of sorafenib both *in vitro* and *in vivo* (Figs 3, 4, 5). Notably, the function of statins was not attenuated under hypoxia, which not only suggests that statins exerted anti-cancer activities in a different manner than the chemotherapeutic agents or the kinase-targeted drugs but also indicates that these drugs can be used in novel strategies to combat the hypoxia-mediated resistance towards sorafenib.

Notably, the cell densities for both the *in vitro* and *in vivo* assays were very high. The reason for this type of cell confluence was to better imitate the intracellular and extracellular environments of advanced HCC tumours. Under these high-density conditions, YAP was predominantly located in the cytoplasmic fraction of normoxic HCC cells, which was consistent with previous studies^45^,^46^. Intriguingly, the hypoxic HCC cells “escape” the cell-cell contact inhibition of the YAP pathway, which may contribute to a variety of hypoxic consequences, including cellular resistance towards sorafenib. Fortunately, the hypoxia-prompted “escape” of YAP suppression was restored by statins, which merits further evaluation in terms of its combination applications with sorafenib in HCC patients.

In summary, the present study showed that YAP activation under hypoxic conditions is a mediator of hypoxia-induced resistance towards sorafenib in HCC cells, which is consistent with the activation of pro-survival and anti-apoptotic genes induced by YAP under hypoxia. We found that statins could abolish hypoxia-activated YAP, thus reversing the hypoxic resistance and increasing the susceptibility of hypoxic HCC cells to sorafenib. Based on these observations, our results provide a rationale for combining statins with sorafenib, under hypoxia, in the treatment of HCC tumours. Additionally, the expression level of YAP might be regarded as a potential biomarker for the early predication of sorafenib resistance, and for these patients, a combination with statins may improve the clinical outcome.

## Methods and Materials

### Cell lines and cell culture

Three hepatocellular carcinoma cell lines were used. HepG2 cells were maintained in DMEM (Gibco, Grand Island, NY, USA) containing 10% heat-inactivated foetal bovine serum (FBS, Gibco, Grand Island, NY, USA). Bel-7402 and SMMC-7721 cells were cultured in RPMI 1640 medium (Gibco, Grand Island, NY, USA) containing 10% FBS(Gibco, Grand Island, NY, USA). All three cell lines were purchased from the Institute of Cell Biology(Shanghai, China), and they were incubated at 37 °C in a 5% CO_2_ atmosphere. Cells were exposed to hypoxic conditions (1% O_2_) in a hypoxia incubator filled with a mixture of 1% O_2_, 5% CO_2_ and 94% N_2_.

### Transfection and siRNA

HCC cells in 6-well plates were transfected 24 h after using X-tremeGENE HP DNA transfection reagent (Roche). The siRNA sequence was purchased from GenePharma Co. (Shanghai, China). The sequences of siRNA were as follows: YAP: 5′-r(GACAUCUUCUGGUCAGAGA)d(TT)-3′; 5′-r(UCUCUGACCAQGAAGAUGUC)d(TT)-3′. The transfection was performed using oligofectamine (Invitrogen) according to the manufacturer’s recommendations.

### Western blot

Whole-cell extracts from cultured cells or tissues were prepared and subjected to western blotting. Equal amounts of cell extracts were resolved by 10% SDS-PAGE, analysed by immunoblotting and visualized by enhanced chemiluminescence (ECL, Amersham Biosciences, Castle Hill, Australia). The following antibodies were used: anti-YAP (Cell Signaling Technology, 4912s), phospho-YAP (Ser127) (Cell Signaling Technology, #4911), β-actin (Santa Cruz, sc-1615), α-tubulin (Santa Cruz, sc-8035), PARP (Santa Cruz, sc-7150), Bcl-xl (Cell Signaling Technology, #2762s), CTGF(Abcam, ab6992), and Cyr61 (Gentex, GTX50042).

### qRT-PCR

Total RNA was extracted from cells or tumours by TRIzol (Invitrogen), and one microgram of total RNA from each sample was used for cDNA synthesis. The quantitative real-time RT-PCR analysis was performed by SYBR (Bio-Rad,172-5124). PCR amplification was conducted using the following primers:

AREG sense: 5′-TTCATGGCGAATGCAATGCAGATACA-3′

AREG antisense: 5′-ATCCGAAAGCTCCACTTCCT-3′

Cyr61 sense: 5′-TCCCTGTTTTTGGAATGGAG-3′

Cyr61 antisense: 5′-TGGTCTTGCTGCATTTCTTG-3′

CTGF sense: 5′-TTTGGCCCAGACCCAACTAT-3′

CTGF antisense: 5′-GTGCAGCCAGAAAGCTCAAA-3′

Bcl-xl sense: 5′-TCCTTGTCTACGCTTTCCACG-3′

Bcl-xl antisense: 5′-GGTCGCATTGTGGCCTTT-3′

β-actin sense: 5′-GGTCATCACTATTGGCAACG-3′

β-actin antisense: 5′-ACGGATGTCAACGTCACACT-3′

### Cell proliferation assay

HCC cells were treated with the compounds at various doses for 48 h in normoxic or hypoxic condition, and cell proliferation was measured by a sulforhodamine B (SRB) protein assay (Sigma, S1402)^47^. Cells (3000/well) were incubated with 10% trichloroacetic acid(TCA) for 1 h (4 °C) and then stained with sulforhodamine B for 20 min. The sulforhodamine B was washed away with 1% glacial acetic acid, and 100 μl of 1% Tris-base was added to each well. The optical density (OD) was determined at 515 nm by a Multiskan Spectrum plate reader (Thermo Electron Corporation, Marietta, OH, USA).

### Immunohistochemical assay

Tissue microarray slides were obtained from US Biomax (BC03119a, Alenabio, Xian, China). Paraffin-embedded tissue microarrays were dewaxed, rehydrated, and subjected to microwaving with sodium citrate buffer (pH 6.0) for YAP (Cell Signaling Technology, 4912s) staining. Then, an SP Kit (HSP0008) was used according to the manufacturer’s recommendations.

### Xenograft tumour growth

Nude mice (5–6 weeks of age) were obtained from Shrek (Shanghai, China). When HepG2 xenograft tumours reached 100 mm^3^, mice received sorafenib(Aladdin, S125098, 30 mg/kg), atorvastatin (Aladdin, A121956, 100 mg/kg) or both of them orally once daily for 15 days. Other mice were orally administered normal saline as controls for 15 days. The volume of the xenograft tumours was estimated using the following formula: tumour volume = 0.5× (major axis) × (minor axis)^2^.

Statements of Ethical Approval: The protocols for the animal study were approved by the Animal Research Committee at Zhejiang University, with ethical approval number IACUC-15003, and all experimental protocols were conducted in accordance with institutional guidelines.

### Immunofluorescence staining

Cells were treated with DMSO or atorvastatin (10 μM) in hypoxic conditionsfor 24 h. Tumourcryostat sections were obtained after harvesting the tumours. Cells or cryostat sections were fixed using 4% paraformaldehyde, and YAP (Santa Cruz, sc-15407) antibody was used, followed by Alexa Fluor-488 staining. Nuclei were observed by staining with DAPI.

### Flow cytometry

For measurement of apoptotic cells by flow cytometry (sub-G_1_), HCC cells were treated with DMSO, sorafenib, atorvastatin or both at the indicated doses in hypoxic condition for 48 h. Cells were then harvested, washed with PBS, and fixed with precooled 70% ethanol at 4 °C. Cells were stained in PBS containing 40 μg/mL RNase A and 10 μg/mL propidium iodide (Sigma, St. Louis, MO) in the dark for 30 min. The percentage of apoptotic cells was shown by cell-cycle distribution using flow cytometry.

### Statistical analyses

The results are expressed as the mean ± SD of at least 3 independent experiments. Differences between two means were analysed by Student’s t-test and were considered statistically significant when *P* < 0.05.

## Additional Information

**How to cite this article**: Zhou, T.-y. *et al*. Inactivation of hypoxia-induced YAP by Statins overcomes hypoxic resistance toSorafenib in hepatocellular carcinoma cells. *Sci. Rep.* **6**, 30483; doi: 10.1038/srep30483 (2016).

## Supplementary Material

Supplementary Information

## Figures and Tables

**Table 1:** Correlation of YAP Expression and Liver Cancer in the Oncomine Online Database.

	Fold Change	p-Value	Overexpression of Gene Rank	Database(Case Number)
Upregulation of YAP in Liver Cancer(Clinical Feature)				
Liver Cancer Type: Liver Cancer Precursor	1.586	<0.0001	in top 4%	Mas Liver(115)
Hepatocellular Carcinoma vs. Normal	1.832	<0.0001	in top 9%	Roessler Liver(43)
Hepatocellular Carcinoma vs. Normal	1.608	<0.0001	in top 19%	Roessler Liver 2(445)
Upregulation of YAP in Liver Cancer(Cell lines)				
Cancer Type: Liver Cancer	2.96	<0.0001	in top 1%	Wooster Cell Line(318)
Cancer Type: Liver Cancer	3.589	<0.0001	in top 1%	Barretina Cell Line(917)
Cancer Type: Liver Cancer	2.139	<0.0001	in top 1%	Garnett Cell Line(732)
