# Supplementary material for: Inactivation of hypoxia-induced YAP by statins overcomes hypoxic resistance tosorafenib in hepatocellular carcinoma cells
Source: Sci Rep. 2016 Aug 1;6:30483. doi: 10.1038/srep30483 (PMC4967870; doi:10.1038/srep30483)

Inactivation of hypoxia-induced YAP by Statins overcomes hypoxic resistance of Sorafenib in hepatocellular carcinoma cells

Tian-yi Zhou[1], Lin-hanZhuang[1], Yan Hu[1], Yu-lu Zhou[1], Wen-kai Lin[1],Dan-dan Wang[1], Zi-qian Wan[1], Lin-lin Chang[1], Ying Chen[1], Mei-dan Ying[1], Zi-bo Chen[1], Song Ye[2], Jian-shu Lou[1], Qiao-jun He[1], Hong Zhu*[,1], Bo Yang*[,1]

[1]Zhejiang Province Key Laboratory of anti-cancer Drug Research, Institute of Pharmacology and Toxicology, College of Pharmaceutical sciences, Zhejiang University, Hangzhou, China

[2]First Affiliated Hospital, School of Medicine, Zhejiang University, Hangzhou, China

*: Correspondence to:

Dr. Hong Zhu, Zhejiang Province Key Laboratory of Anti-Cancer Drug Research, College of Pharmaceutical Sciences, Zhejiang University, 866#Yuhangtang Rd, Hangzhou, Zhejiang 310058, China, hongzhu@zju.edu.cn;

Dr. Bo Yang, Zhejiang Province Key Laboratory of Anti-Cancer Drug Research, College of Pharmaceutical Sciences, Zhejiang University, 866#Yuhangtang Rd, Hangzhou, Zhejiang 310058, China, yang924@zju.edu.cn;

Keywords: hypoxia, resistance, sorafenib, YAP, hepatocellular carcinoma cells

Supplemental Materials and Methods:

Transfection of TAZ siRNA

The siRNA sequence was purchased from GenePharma Co. (Shanghai, China). The sequences of siRNA were as follows: TAZ: 5'-r(GGCCAGAGAUAUUUCCUUA)d(TT)-3'; 5'-r(UAAGGAAAUAUCUCUGGCC)d(TT)-3'. The transfection was performed using oligofectamine (Invitrogen) according to the manufacturer's recommendations.

Supplemental Figure Legends:

Figure S1: TAZ depletion by siTAZ failed to abrogate sorafenib resistance under hypoxia in HepG2.

Figure S2: HepG2 cells were treated with sorafenib at various serial concentrations (48 h) in the presence or absence of pravastatin. "Heat" graphs were presented to displayed the combination effects. Z-axis: Inhibition ratio of treated groups under hypoxia (1%O_2) or normoxia (20%O_2); X-axis: the concentrations of pravastatin; Y-axis: the concentration of sorafenib. And the colors representative for the inhibition ratio were presented.

Figure S3: The combination of atorvastatin with sorafenib increased the PARP cleavage, accompanied with the amelioration of hypoxia-activated YAP pathway. (A) The levels of PARP and cleaved-PARP in Bel-7402 and SMMC-7721 cells were detected by western blot analysis. Cells were treated with sorafenib (10 μM) or atorvastatin (10 μM) alone or both for 48 h. The levels of p-YAP was also monitored in those groups treated for 24 h. (B) qRT-PCR analysis of the YAP target genes *Bcl-xL, CTGF* and *Cyr61* in HepG2 cells exposed to hypoxia (1%O_2) or normoxia (20%O_2) for 24 h. Data are representative of 3 independent experiments and are expressed as the mean ± SD.

Figure S3: YAP cellular localization in the HCC patient samples on the tissue microarray.

Figure S1

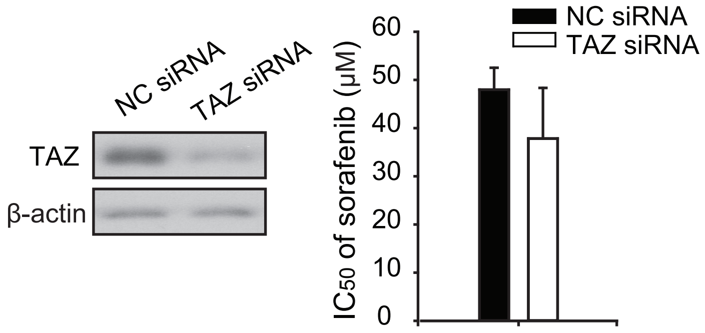

Figure S2

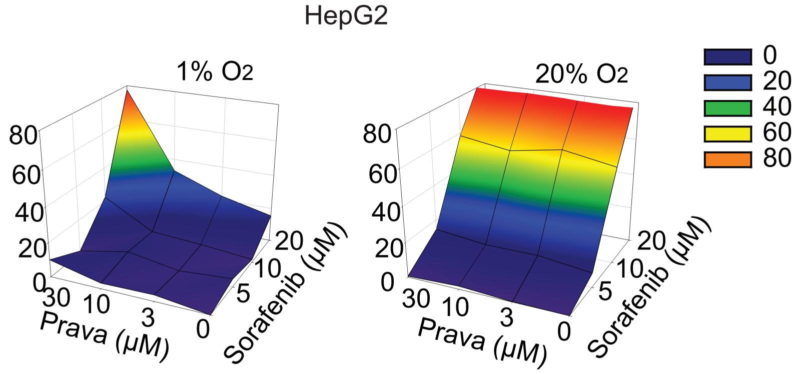

Figure S3

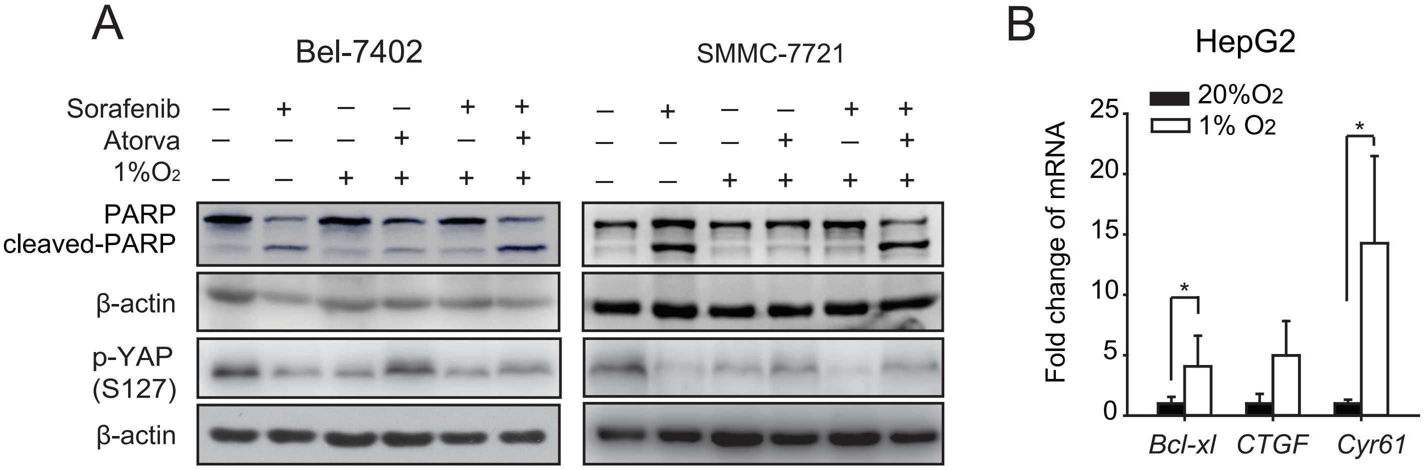

Figure S4

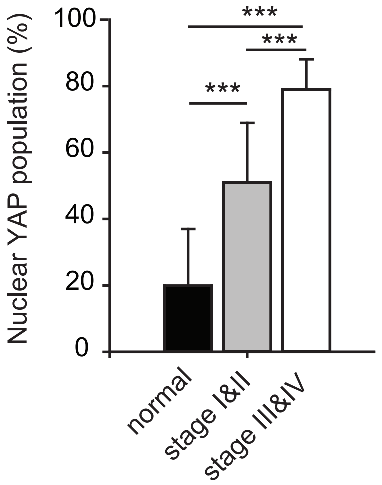

Supplement: Supplementary Information [file srep30483-s1.pdf]
